# Primary dementia care based on the individual needs of the patient: study protocol of the cluster randomized controlled trial, DemStepCare

**DOI:** 10.1186/s12877-021-02114-z

**Published:** 2021-04-01

**Authors:** Isabella Bablok, Harald Binder, Dominikus Stelzer, Klaus Kaier, Erika Graf, Julian Wangler, Michael Jansky, Michael Löhr, Michael Schulz, Marie Kockläuner, Katharina Geschke, Alexandra Wuttke-Linnemann, Andreas Fellgiebel, Erik Farin, study group DemStepCare

**Affiliations:** 1 Institute of Medical Biometry and Statistics, Section of Health Care Research and Rehabilitation Research, Faculty of Medicine and Medical Center, University of Freiburg, Freiburg, Germany; 2 Institute of Medical Biometry and Statistics, Faculty of Medicine and Medical Center, University of Freiburg, Freiburg, Germany; 3 Center for General and Geriatric Medicine, University Medical Center Mainz, Johannes Gutenberg University, Mainz, Germany; 4 Landschaftsverband Westfalen-Lippe, Hospital Gütersloh, Gütersloh, Germany; 5 Diakonie University of Applied Sciences, Bielefeld, Germany; 6 Department of Pharmacy, University Medical Center Mainz, Johannes Gutenberg University, Mainz, Germany; 7 Department of Psychiatry and Psychotherapy, University Medical Center Mainz, Johannes Gutenberg University, Mainz, Germany; 8 Center for Mental Health in Old Age, Landeskrankenhaus (AöR), Mainz, Germany

**Keywords:** Dementia, Primary care, Case management, Risk assessment, Multi-professional team, Caregivers, Evaluation, Cluster randomized controlled trial

## Abstract

**Background:**

Most people with dementia (PwD) are cared for at home, with general practitioners (GPs) playing a key part in the treatment. However, primary dementia care suffers from a number of shortcomings: Often, diagnoses are made too late and therapies by GPs do not follow the guidelines. In cases of acute crises, PwD are too often admitted to hospital with adverse effects on the further course of the disease.

The aim of this study is to implement and evaluate a new GP-based, complex dementia care model, DemStepCare. DemStepCare aims to ensure demand-oriented, stepped care for PwD and their caregivers.

**Methods/design:**

In a cluster randomized controlled trial, the care of PwD receiving a complex intervention, where the GP is supported by a multi-professional team, is compared to (slightly expanded) usual care.

GPs are clustered by GP practice, with 120 GP practices participating in total. GP practices are randomized to an intervention or a control group. 800 PwD are to be included per group. Recruitment takes place in Rhineland-Palatinate, Germany. In addition, a second control group with at least 800 PwD will be formed using aggregated routine data from German health insurance companies. The intervention comprises the training of GPs, case management including repeated risk assessment of the patients’ care situation, the demand-oriented service of an outpatient clinic, an electronic case record, external medication analyses and a link to regional support services. The primary aims of the intervention are to positively influence the quality of life for PwD, to reduce the caregivers’ burden, and to reduce the days spent in hospital. Secondary endpoints address medication adequacy and GPs’ attitudes and sensitivity towards dementia, among others.

**Discussion:**

The GP-based dementia care model DemStepCare is intended to combine a number of promising interventions to provide a complex, stepped intervention that follows the individual needs of PwD and their caregivers. Its effectiveness and feasibility will be assessed in a formative and a summative evaluation.

**Trial registration:**

German Register of Clinical Trials (Deutsches Register Klinischer Studien, DRKS), DRKS00023560. Registered 13 November 2020 - Retrospectively registered. HTML&TRIAL_ID=DRKS00023560.

## Introduction

### Background and rationale

Approximately 50 million people worldwide live with dementia [1, 2]. By 2050, this number is expected to have tripled [3]. When dealing with dementia, outpatient care is of great importance: The majority of people with dementia (PwD) are cared for by family members [4]. However, primary dementia care still faces a number of problems.

#### Provide timely and accurate diagnoses

Some of the problems can be ascribed to general practitioners (GPs), who should often be the first to detect the onset of dementia and deal with it adequately. It has been shown that GPs are often reluctant to face the diagnosis of dementia and do not follow the existing guidelines [5–9]. GPs do not want to “scare off” their patients and they feel that they have little chance to do anything about the disease [10]. Affective factors and experienced self-efficacy are of great importance here, not just the GPs’ previous knowledge and competence [11, 12]. As a result, dementia diagnoses are often not made [13, 14] or only when home care is already at risk. From a therapeutic perspective, however, it has been shown that early diagnosis of dementia is crucial to positively influence the course of the disease [15–17]. A major goal in improving primary dementia care is therefore the timely and accurate diagnosis of dementia.

#### Avoid (medical) errors in therapy

However, not only the diagnostic process needs to be improved. It has been shown that GPs are often overburdened or succumb to a number of misconceptions when it comes to the treatment of dementia [18, 19]. In many cases, the existing guidelines [20, 17] are not followed [21]. For example, there are often errors in the prescription of medicines [22–25]. One difficulty here is that PwD are at higher risk of multimorbidity and polymedication due to their usually older age [26]. Dementia-specific training courses for GPs might be beneficial here [27, 28].

#### Involve and coordinate various healthcare professionals

Other problems of primary dementia care are structural in nature. The complexity of the clinical picture necessitates multi-professional and multimodal care [29, 30]. Needs vary greatly between individuals and over the course of the disease [31], making individualized and stepped interventions necessary. Integrated, cross-sectoral approaches to effective outpatient dementia care are needed [32]. GPs often lack knowledge about existing regional services to support PwD and their caregivers [33]. Studies on case management in dementia care have not yet revealed consistent results [34], but case management seems to be a promising factor that might facilitate the needs-based [35], stepped activation of different healthcare professionals and thus contribute to improving the care of PwD [36].

Furthermore, the different healthcare professionals involved must communicate effectively with each other to ensure continuity of care. There is a lack of efficient (digital) communication structures, especially in rural areas [37]. User-friendly solutions are needed here.

#### Support informal caregivers and avoid hospitalization

Family caregivers are exposed to increased physical and psychological stress [38–41]. This in turn can affect the caregivers’ health [41, 38]. If any additional crises occur, this can lead to hospitalization of the PwD they care for [42]. However, it has been shown that hospitalization of PwD tends to have negative consequences for their health [43] as well as for the caregivers’ emotional stability [44]. In addition, hospital stays are expensive [45] and burden the healthcare system. It is therefore an important objective to avoid unnecessary hospitalization [46], while providing effective support for caregivers.

### Objectives

The study is intended to bundle a number of possibly effective interventions to provide a complex, stepped intervention that follows the individual needs of PwD and their caregivers. DemStepCare seeks to provide structures that improve and stabilize outpatient dementia care. The primary aims of the intervention are as follows: to positively influence the quality of life of PwD, to reduce the caregivers’ burden, and to reduce the days spent in hospital.

Further goals include the improvement of guideline-based diagnostics and therapy, improved safety and quality of drug therapy and increased sensitivity to the topic of dementia, as well as an increased willingness and acceptance of GPs to provide care for PwD. In DemStepCare regional support services are to be used more effectively, risk constellations in home care are to be detected earlier and home care is to be stabilized. In addition, it will be evaluated whether the intervention is cost effective. A process evaluation will be conducted to investigate how the intervention works and to identify factors that hinder its implementation.

### Trial design

In a prospective, cluster randomized trial, two types of primary dementia care will be compared to each other: the innovative complex intervention DemStepCare (intervention group) and a slightly expanded usual care condition (control group 1). In addition, a second (historical) control group will be formed, which will be composed of aggregated routine data from health insurance companies involved in the project (control group 2).

A total of 120 GP practices are to be included for the intervention group and control group 1. Randomization with a 1:1 ratio is undertaken at the level of GPs’ practices and stratified by dementia sensitivity of the GPs. 800 PwD are to be included per group (i.e. intervention group, control group 1, control group 2).

Measurement times for PwD and caregivers are t0 (=time of inclusion) and t1 (=minimum duration of the intervention / nine months after t0). In addition, in the intervention group the further course of treatment is examined longitudinally and included in the evaluation (t2 = end of intervention / max. 27 months after t0).

**Fig. 1:**
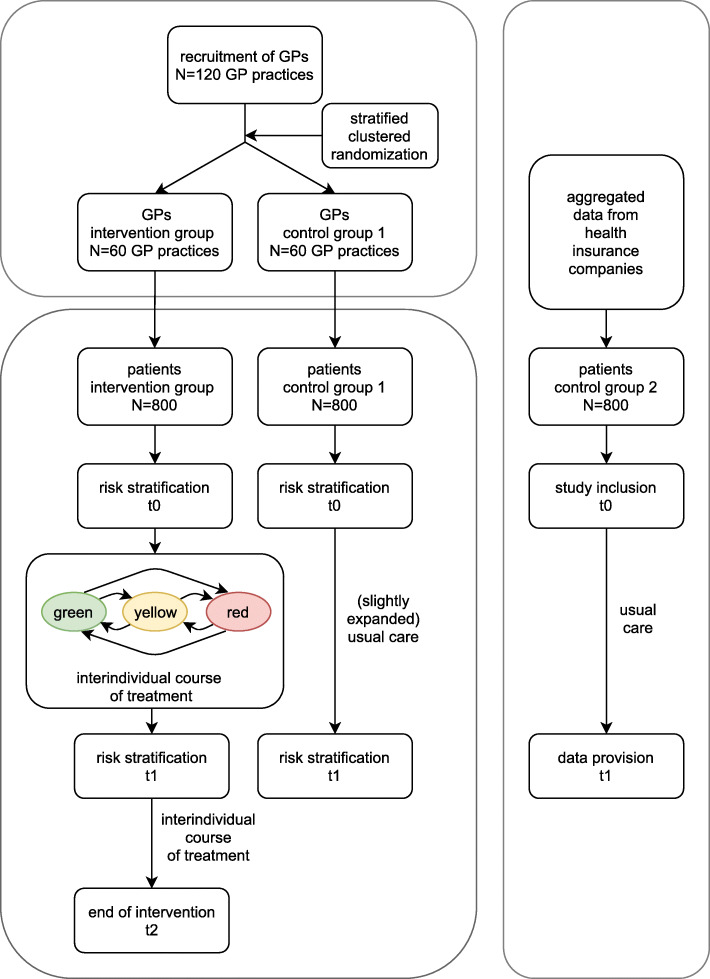
Design of the cluster randomized controlled study DemStepCare. t0 = study inclusion, t1 = minimum duration of the intervention, nine months after t0; t2 = end of intervention, max. 27 months after t0

The design of the study is shown in Fig. 1.

## Methods: participants, interventions, and outcomes

### Study setting

The catchment area of DemStepCare is in a largely rural area of Rhineland-Palatinate, Germany. The outpatient clinic is located in the middle of the recruitment area at Rheinhessen Fachklinik Alzey, Landeskrankenhaus (AöR), allowing all patients of the intervention group to be reached.

Control group 2 will be composed of patients from all over Germany.

### Eligibility criteria

Patients and (if possible) their caregivers are eligible to participate in the intervention group or control group 1, if they meet the following criteria:

- Diagnosis of dementia
- Place of residence within the study area
- Informed consent to participate in DemStepCare

Living in a nursing home is defined as an exclusion criterion.

As patients can only be included via their GPs, the patients must be cared for by a GP who participates in DemStepCare. The only requirement for participating GPs is that their practice must be within the study area.

### Interventions

#### Description of the intervention condition

The first part of the intervention involves training of the GPs: GPs are to identify dementias earlier and become more confident in dealing with the diagnosis. The training courses impart knowledge about the appropriate diagnosis and treatment of PwD following the guidelines of the German Society for Psychiatry and Psychotherapy, Psychosomatics and Neurology (Deutsche Gesellschaft für Psychiatrie und Psychotherapie, Psychosomatik und Nervenheilkunde, DGPPN) [17].

The so trained GPs enroll patients and if possible their caregivers in the study program. Patients with a pre-existing dementia diagnosis as well as patients who receive a new dementia diagnosis while DemStepCare is running can be included in the study. If there is a suspected dementia, the GPs can either diagnose dementia themselves or refer the patients to a specialist / memory clinic. Patients with a confirmed dementia diagnosis and if possible their caregivers are informed about the study and asked if they are willing to participate. If patients and their caregivers wish to participate, they must give their written consent. If there is a legal guardian, the legal guardian must give written consent in addition to the patient. All patients require a basic competency to consent, otherwise they cannot participate. Having given their written consent, patients are assigned to a case manager who conducts a first counselling interview, including information on regional support options (e.g. a supervised relatives’ group that is part of DemStepCare, counselling centers) and advice on care. At this first encounter, the case manager assesses the individual risk constellation of the home care situation using a dementia specific screening tool (in German: Demenzspezifisches Screening zur Versorgungssituation, DSV [47]). The DSV addresses both aspects concerning the patient and aspects concerning the caregiver. When evaluating the DSV, there are two possible results: green (stable situation) or yellow (increased risk). In the case of an increased risk, two further tests are to follow, the CMAI, which measures agitated behavior [48, 49], and the NOSGER, which covers clinically relevant behavior of psychiatric disorders of old age [50, 51]. Based on the results of these tests, it is decided whether the case can be assessed as yellow or whether it is a red case (acute crisis).

Depending on the respective category, the case manager proceeds differently: In green cases, the case manager offers information on regional support options and sets a next appointment for six months later. In yellow cases, the case manager provides more intense care contacting the patient at least once a month. In red cases, the case manager calls in the outpatient clinic that consists of specialized nursing staff, a psychiatrist, and a social worker. The outpatient clinic continues to provide care until the crisis is overcome. To this end, the outpatient clinic offers home visits of the appropriate frequency to avoid hospitalization. If it is mandatory, an inpatient stay in a geriatric psychiatric clinic is initiated.

**Fig. 2:**
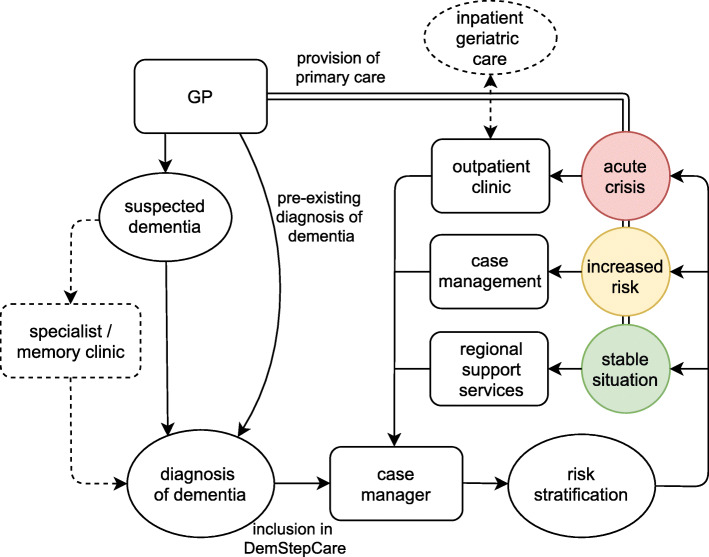
Overview of the DemStepCare intervention (medication analyses by the pharmacy and communication via the electronic case record are not shown to ensure clarity)

Throughout the course of DemStepCare, this risk stratification is regularly repeated (at least at t0, t1, and t2, and invididually if needed) and appropriate action is taken to ensure that each patient receives the care he or she needs. Figure 2 shows an overview of the described procedure.

In addition, clinical pharmacists regularly conduct structured medication reviews with the aim of improving the quality and safety of the drug therapy. The attending GPs are alerted to inappropriate medication and drug-related problems (DRP).

Communication between the different parties (i.e. GPs, case managers, outpatient clinic, pharmacy) takes place via a specifically adapted electronic case record. Thus, timely and effective communication is facilitated.

While participating in DemStepCare, patients continue to be cared for by their GPs, who can repeatedly take part in dementia-specific training sessions as the project progresses.

#### Description of control condition 1

GPs assigned to the control group 1 receive a shorter form of training. A case manager is employed and risk stratification is conducted, too, but the result has no influence on the further treatment of the patients, i.e. it only serves evaluative purposes. Neither the GPs nor the patients get to know the result of the stratification. There is a follow-up meeting with the case manager after nine months.

As there are small elements of intervention in control condition 1, control condition 1 cannot be considered as being merely usual care. However, the training of the GPs is intended as an incentive and the visits of the case managers are necessary to gather data for the evaluation. We therefore decided to use this slightly expanded usual care condition as control condition 1. Control group 2, the historical control condition, is inherently usual care.

Training of the GPs in intervention as well as in control condition 1 is carried out by experienced healthcare professionals. The case managers also receive training resulting in a certification as case manager according to the German Society for Care and Case Management (in German: Deutsche Gesellschaft für Care und Case Management, DGCC).

## Outcomes

The evaluation comprises a formative process evaluation and a summative evaluation. We thereby follow the recommendations for evaluating complex interventions [52]. Both types of intervention are aimed at both people who receive the DemStepCare intervention (patients and their caregivers), and people who provide the DemStepCare intervention (GPs and case managers). There are different points of measurements for different participants.

Due to the coronavirus pandemic, the delivery of intervention had to be suspended in April and May 2020. During this time, a nationwide lockdown was implemented in Germany, which applied from mid-March 2020 and was then successively lifted. In April and May 2020, participants of DemStepCare had access to a DemStepCare telephone helpline. No other elements of the intervention were offered for the safety of the patients. For this reason, it was decided to shift some points of measurement: If a person has undergone the two-month intervention pause, the t1-measurement will also take place two months later than originally planned (e.g., 11 months after t0 instead of nine months after t0 for PwD and caregivers).

### Process evaluation

Process evaluations aim at monitoring the feasibility and perceived success of the evaluated interventions. Relevant processes are identified, described and reported back to the involved parties.

The main questions of the process evaluation concern acceptance of, satisfaction with, and a benefit assessment of DemStepCare. Following a mixed-methods approach, data from caregivers and healthcare providers are collected via questionnaires and interviews. Patients are solely interviewed due to the assumed cognitive impairment and the associated stress possibly resulting from a written survey.

For the caregivers, an adapted version of the Perceived Involvement in Care Scale (PICS) [53, 54] is used to assess interaction with healthcare providers and involvement in care. In addition, new items will be developed in order to be able to precisely evaluate the satisfaction with the DemStepCare intervention. This survey takes place at baseline (t0) and at t1. A random sample of caregivers and patients (*N* = 20) will be additionally interviewed. The interviews will follow a semi-structured interview guide. Questions asked during the interviews will include “Were there situations or processes in DemStepCare that you experienced as difficult or worth improving?”; “How has your situation changed due to DemStepCare?” The interviews will be conducted at t1.

As the case managers are trained specifically for the project and become active in a new professional field, it will be assessed how their professional coping behavior, their stress management ability and their sense of coherence develop. Validated scales are used for this purpose: a scale to assess work-related behavior and experience patterns (in German: Arbeitsbezogenes Verhaltens- und Erlebensmuster, AVEM [55]), the Stress Management Questionnaire (in German: Stressverarbeitungsfragebogen, SVF [56]), and a scale to assess the sense of coherence (SOC) [57]. There are no English versions of the AVEM and SVF. The AVEM covers health-promoting or health-damaging behavior associated with professional requirements. This behavior is assigned to four patterns: health, protection, risk of overstraining oneself, and risk of chronic exhaustion and resignation. The SVF addresses different ways of coping with or processing stressful events (e.g. avoidance, distraction, and the need for social support). The SOC measures the extent to which people understand, manage and attribute meaning to what happens around them. There will be three measurements: one at baseline, i.e. before the case managers start work (t0_CM_), one after 12 months (t1_CM_), and one at the end of the intervention phase (t2_CM_). In addition, all the case managers are to be interviewed. The interviews will follow a semi-structured interview guide. Questions asked during the interviews will include “How do you experience the current care situation of your patients and their families?”; “How do you experience the collaboration with the GPs?” The interviews will start 20 months after the start of the intervention.

All GPs are interviewed in a panel survey. The questions for the survey were specifically developed for DemStepCare drawing on findings from our preliminary studies [58, 10, 59]. They contain both closed-ended and open-ended questions asking about satisfaction or dissatisfaction with different aspects of DemStepCare. There will be three measurements: one at baseline, i.e. before randomization and the first training session of the GPs (t0_GP_), one 12 months after completion of the first training (t1_GP_), and one at the end of the intervention phase (t2_GP_). A random sample of GPs (*N* = 10) will be additionally interviewed. The interviews will follow a semi-structured interview guide. Questions asked during the interviews will be similar to those used for the case managers, except that GPs will be asked about the collaboration with the case managers. The interviews will be conducted at t1_GP_.

### Summative evaluation

The summary evaluation examines the primary endpoints (i.e., quality of life of PwD, caregiver burden, and days spent in hospital) and various secondary endpoints, which are described below.

In order to compare the intervention group and control group 1, these two groups are examined at t0 (baseline) and t1 (=minimum duration of the intervention, 9 months after t0). In the intervention group, the intervention will also continue to be offered throughout the treatment period until t2 (= end of intervention; max. 27 months), so that the individual treatment courses in the intervention group can be further investigated. The intra-individual and longitudinal analyses in this group allow the predicting of the effectiveness of the treatment more precisely.

#### Primary endpoints

The three primary endpoints are assessed via questionnaires. An overview is shown in Table 1.

**Table 1:** Primary endpoints

Construct	Respondents	Instrument at a given time		Group comparison
		t0	t1	
Quality of life (patients)	Patients and caregivers IG+ CG1	Quality of Life in Alzheimer’s Disease (QOL-AD) [60]	QOL-AD	IG vs. CG1
Caregiver burden (caregivers)	Caregivers IG + CG1	Berlin Inventory of Caregivers’ Burden with Dementia Patients (in German: Berliner Inventar zur Angehörigenbelastung – Demenz, BIZA-D) [61]	BIZA-D	IG vs. CG1
Days spent in hospital (patients)	Patients and caregivers IG + CG1	Original item	Original item	IG vs. CG1

*IG* intervention group, *CG1* control group 1, t0 = study inclusion, t1 = minimum duration of the intervention / nine months after t0

To measure the patients’ quality of life, we use the Quality of Life in Alzheimer’s Disease (QOL-AD), a validated scale developed specifically for PwD [60]. The QOL-AD encompasses 13 areas of life that need to be evaluated (e.g. family, living conditions, energy). There is a version for patients and a proxy version. In DemStepCare, we use both versions, with the proxy version being filled in by the caregivers. Both scores are integrated into a total score. There is one baseline measurement (t0) and one measurement at t1.

To measure the caregiver burden, we use the short form of the Berlin Inventory of Caregivers’ Burden with Dementia Patients (in German: Berliner Inventar zur Angehörigenbelastung – Demenz; BIZA-D) which was specifically designed and validated to measure the burden resulting from caring for a person with dementia [61]. The BIZA-D is a comprehensive instrument that, among other things, queries various care tasks and associated burdens, the social support experienced, and acceptance of the situation. Caregivers fill in the BIZA-D at baseline (t0) and at t1.

To measure the days spent in hospital, we use a self-developed item at t0 and t1. We ask for days spent in hospital in the last nine months. We use one version for patients and one proxy version for caregivers. In addition, we will receive individual level data from the participating health insurance companies for the inpatient treatment days. Since only a part of the participants will presumably be insured with these health insurance companies, we will use this data for plausibility checks (comparison self-report vs. claim data). The main data basis for the primary endpoint will be provided by the questionnaires.

#### Medication analyses

Within the multi-professional concept, clinical pharmacists conduct structured medication reviews at t0, t1 and t2. In the intervention group, additional medication analyses are performed quarterly and in case of crisis.

The quality of the prescribed medication is primarily measured by the Medication Appropriateness Index (MAI), an implicit prescriptive quality measure with good inter-rater and intra-rater reliability [62, 63]. The index provides ten criteria relevant for appropriate prescription, formulated as questions regarding indication, efficacy, dosage, correct dosage regimens, practicable directions for use, drug-drug interactions, drug-disease interactions, duplicate prescriptions, treatment duration and cost [62].

For a standardized evaluation of the criteria by the evaluators, the MAI-Score provides general instructions for the use of the index, precise definitions for each criterion and specific instructions and examples for answering the ten questions [62].

The evaluation of the criteria results in a weighted point score that represents the adequacy of each prescribed drug [63]. Only nine of the ten criteria of the MAI are used in DemStepCare, the question of economic efficiency of the prescription has been excluded.

In addition to the MAI, the following aspects are also addressed within the medication review to assess and evaluate safety and quality of medication:

- underuse of medication for diseases requiring therapy using the START criteria [64]
- unsuitable drugs for geriatric patients based on the PRISCUS-list [65], FORTA-list [66] and STOPP criteria [64]
- reduction of multimedication using defined deprescribing strategies
- the number of drug-related problems according to PCNE version 9.0

The required information is retrieved from the electronic case record. Available clinical data (diagnoses, laboratory values) are included in the analysis. The implementation rate of pharmaceutical recommendations in the intervention group is determined based on the update of patient records.

#### Risk stratification of home care constellation

The regular assessment of risks in home care is a central component of the DemStepCare intervention, since the further course of treatment in the intervention group is based on the results of this assessment. The DSV is used for this purpose [47]. Its data concerning stability of care will also be integrated as a secondary endpoint in the evaluation.

#### Hospitalization

Besides the primary endpoint “days spent in hospital” (intervention group vs. control group 1), two additional secondary endpoints are related to hospital stays: Firstly, a comparison of days spent in hospital is also made between the intervention group and control group 2. For this purpose, the health insurance companies involved in the project provide data for control group 2. Secondly, the number of hospital stays is also analyzed (without considering the length of each stay), both between the intervention group and control group 1 and between the intervention group and control group 2. As with the hospital days, data is available either via questionnaires (intervention group and control group 1) or from aggregated data from the health insurance companies (control group 2) as well as for a small percentage of individual data from the health insurance companies (intervention group and control group 1).

#### Secondary endpoints relating to caregivers

The well-being of caregivers plays a major role in DemStepCare. Therefore, some secondary endpoints also refer to the caregivers. Quality of life is measured with the WHOQOL-Bref, a comprehensive instrument of the WHO, which covers various aspects of this concept [67]. We also use the Perceived Stress Scale (PSS-10), which asks for the perceived stress in the last month [68, 69]. We assess the caregivers’ resilience by using the Brief Resilience Scale (BRS) [70]. Additionally, comorbidities are queried. For this purpose, the KoMo-Score is used [71], to which typical comorbidities known from the care of PwD have been added. Caregivers fill in questionnaires with these scales at baseline (t0) and at t1.

The case managers assess the perceived stress of the caregivers at each interaction (including t0, t1, and t2 in the intervention group) on a 10-point scale.

In addition, the case managers note whether the caregivers use any support services. This aspect is also covered by the caregivers’ self-report in the questionnaires (at t0 and t1).

The secondary endpoints relating to patients and caregivers are shown in Table 2.

**Table 2:** Secondary endpoints relating to patients and caregivers

Construct	Respondents	Instrument at a given time			Group comparison
		t0	t1	t2	
Quality of life (caregivers)	Caregivers IG + CG1	World Health Organization Quality of Life Short Form (WHOQOL-Bref) [67]	WHOQOL-Bref		IG vs. CG1
Comorbidities (caregivers)	Caregivers IG + CG1	Comorbidity Score (KoMo-Score) [71]	KoMo-Score		IG vs. CG1
Stress (caregivers)	Caregivers IG + CG1	Perceived Stress Scale (PSS-10) [69, 68]	PSS-10		IG vs. CG1
	Case managers IG + CG1	10 point scale	10 point scale		
	Case managers IG			10 point scale	
Resilience (caregivers)	Caregivers IG + CG1	Brief Resilience Scale (BRS) [70]	BRS		IG vs. CG1
Stability of care situation (patients and caregivers)	Case managers IG + CG1	Dementia specific screening tool (in German: Demenzspezifisches Screening zur Versorgungssituation, DSV [47])	DSV		IG vs. CG1
	Case managers IG			DSV	
Days spent in hospital (patients)	Patients and caregivers IG	Original item	Original item		IG vs. CG2
	Routine data CG2	Aggregated data from health insurance companies	Aggregated data from health insurance companies		
Hospital admissions (patients)	Patients and caregivers IG + CG1	Original item	Original item		IG vs. CG1 IG vs. CG2
	Routine data IG + CG1	Individual data from health insurance companies	Individual data from health insurance companies	Individual data from health insurance companies	
	Routine data CG2	Aggregated data from health insurance companies	Aggregated data from health insurance companies		
Utilization of outpatient support services (caregivers)	Caregivers IG + CG1	Original item	Original item		IG vs. CG1
	Case managers IG + CG1	Original item	Original item		
Quality and safety of medication (patients)	Pharmacy IG + CG1	Medication review (using the Medication Appropriateness Index (MAI) [63, 62], START criteria [64], PRISCUS-list [65], FORTA-list [66], STOPP-criteria [64], deprescribing strategies, PCNE version 9.0)	Medication review	Medication review	IG vs. CG1

*IG* intervention group, *CG* control group, t0 = study inclusion, t1 = minimum duration of the intervention / nine months after t0, t2 = end of intervention / max. 27 months after t0. The secondary endpoints which refer to the GPs are not shown in this table because there are different measurement times (t0_GP_, t1_GP_, t2_GP_)

#### Secondary endpoints relating to GPs

All participating GPs take part in a panel survey. Here, attitudes, self-efficacy, experienced competence, commitment, and perceived challenges with regard to the diagnosis and treatment of dementia are investigated. We use the term “dementia sensitivity” for this complex, heterogeneous bundle of attitudes and experienced abilities. The questionnaire that we developed especially for DemStepCare is based on our previous experience in this field [10, 58, 59]. In the case of the GPs, there are three measurements: one at baseline (t0_GP_), i.e. before randomization and the first training session of the GPs, one 12 months after completion of the first training (t1_GP_), and one at the end of the intervention phase (t2_GP_).

### Participant timeline

**Fig. 3:**
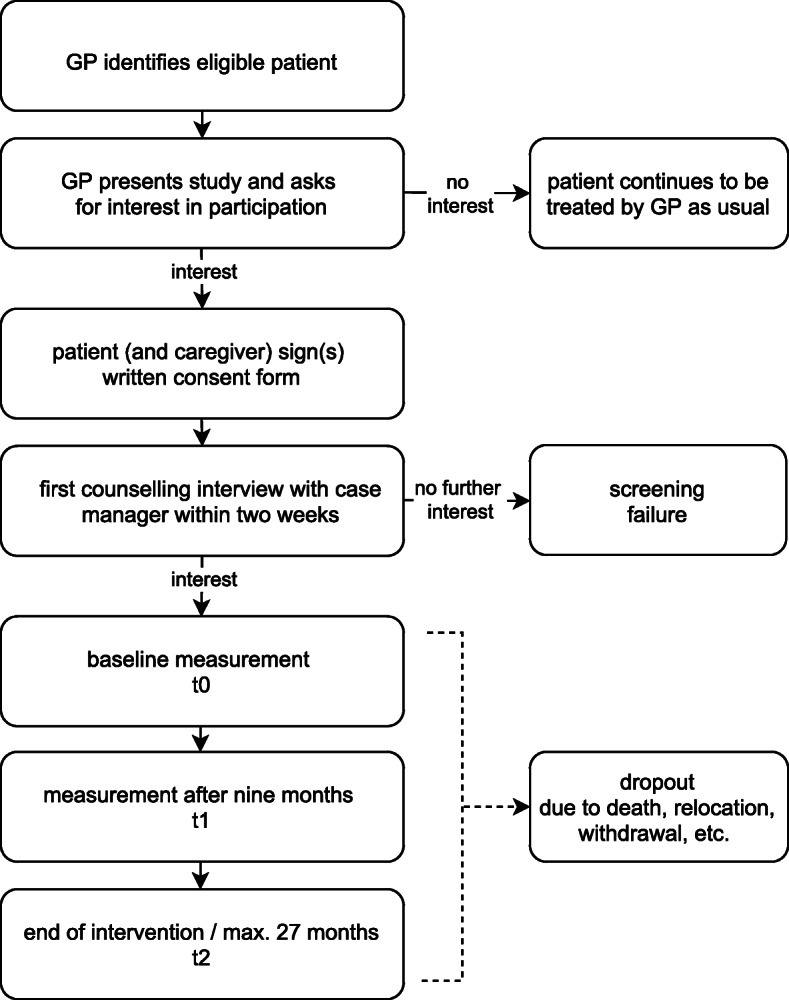
Timeline of DemStepCare

The timeline of DemStepCare is shown in Fig. 3.

### Participants

#### Sample size

The aim is to confirm whether the intervention group performs better than control group 1 with regard to the three primary endpoints. We assume that there are low to medium effects (effect sizes Cohen’s d = 0.30). Following the usual conventions, we require a power of 0.80 and two-sided testing. For the alpha level, a Bonferroni adjustment (5%/3 = 1.67%) is made due to the multiple testing of three primary endpoints. The number of cases required under these assumptions was calculated with the software G*Power.

Due to the clustering by GP practice, we need to calculate the design effect. A value of 0.01 is assumed for the intra-cluster correlation [72]. The number of cases per cluster (patients per practice) is assumed to be *N* = 15. The design effect is therefore DE = 1 + 0.01x (15–1) = 1.14.

Disregarding the clustering, *N* = 235 cases per group would be necessary for statistically significant detection of a difference between the intervention group and control group 1. Thus, taking into account the design effect, 235*1.14 = 268 cases per group with complete data will be required.

It is estimated that 13 to 15 patients per practice will be included, so with 60 participating practices per group there will be approximately *N* = 800 patients per group. Our previous experience with similar study conditions suggests that dropout occurs in about one third of the cases. Assuming a dropout rate of one third, 800*2/3 = 533 persons remain in each patient sample. For the sample of caregivers, we assume a comparable dropout rate, but we must also take into account that only in about two-thirds of all cases is a caregiver available and willing to participate. In the sample of caregivers, 800*4/9 = 355 cases are therefore to be expected. Comparing these numbers with the required number of *N* = 268, there is a buffer in case of unexpected problems during recruitment.

#### Recruitment of participants

#### Recruitment of GPs

We will reach GPs by public advertising, sending out flyers, and advertising at events, as well as face-to-face communication by our project partners.

#### Recruitment of patients and their caregivers

Patients who are potentially eligible to participate in DemStepCare are contacted by their GPs. If possible, their caregivers are also addressed. Those who agree to join the study are assigned to either the intervention or control group 1, based on the group membership of their respective GP.

### Methods: assignment of interventions

Randomization of the GPs is carried out by an independent trust agency. There is clustering at the level of GP practices to ensure that all the GPs in a practice belong to the same group (i.e. intervention group or control group 1). Beforehand, all the GPs fill in a questionnaire about their dementia sensitivity. Here we assess whether GPs experience themselves as self-effective in terms of the diagnosis and therapy of dementia. The dementia sensitivity score is used as a stratifying variable in the randomization process. To this end, dementia sensitivity scores are divided into three categories: low (1), medium (2), and high (3). If the dementia sensitivity categories of GPs in a GP practice differ from each other, a mean value is calculated and rounded up or down to the nearest number. In case this value is in the middle of two numbers, the lower number (i.e. the less sensitive category) is used. The process of stratifying is intended to prevent particularly dementia-sensitive GPs from being in the same group, which could lead to effects that would then be falsely attributed to the intervention.

### Methods: data collection, management, and analysis

All details that specify data protection in the project are documented in a written concept. The team members of DemStepCare are required to adhere to applicable data protection regulations by their respective institutions.

The electronic case record used by the DemStepCare intervention team has a client that can be viewed by members of the evaluation team. Here, addresses are stored that are necessary to contact the participants. No further clinical information is displayed. Once all the data for the evaluation has been collected, this client is immediately deleted.

A multi-factor authentication system (entrust identity guard) is used to ensure that only authorized persons have access to data from the electronic case record. The participating persons first log in to the electronic case record with an individual user name and password. Then a code is generated which has to be entered additionally so that the login can be completed.

All patients automatically receive a pseudonym through the electronic case record, which is used during data collection. The collected data will be forwarded to an independent trust agency. The trust agency assigns new pseudonyms and forwards the data set to the main evaluator. The double pseudonymization is intended to prevent the linking of data from data acquisition and data evaluation.

The main evaluator will distribute smaller data sets to the evaluating institutes. Evaluators who have had access to directly identifiable data (contact data) in the course of data collection commit themselves to deleting all the data related to data collection before receiving an evaluation data set. The only exception to this are the archived paper questionnaires, which are sealed and destroyed after one year.

After 10 years, all electronic data will be deleted.

#### Statistical methods

To evaluate the three primary endpoints, two-sided comparisons between intervention and control group 1 are performed to test for differences with multiple test level alpha = 5% using Bonferroni-Holm adjustment (lowest *p*-value < 1.67%, if applicable second lowest *p*-value < 2.5%, if applicable highest *p*-value < 5%). Due to the typically skewed distribution, the evaluation of the number of days spent in hospital is performed using negative binomial regression. The quality of life of the patients and caregiver burden are investigated using generalized linear regression models with an adjustment for the respective baseline measurements. The multi-level structure of the data is considered in all regression models by means of random effects at the level of the GPs practices. In addition, it is taken into account that some of the participants were affected by a COVID-19-related treatment break. In all three analyses, the dementia sensitivity of the GPs, which is relevant for randomization, is adjusted.

In addition, exploratory analyses are carried out using structural equation models to investigate the secondary hypotheses on how the intervention works.

#### Health economic evaluation

For health economic evaluation, the type, frequency and duration of hospitalization are first used to determine the costs of hospitalization using standardized evaluation rates [73]. Then, the intervention-related cost reduction (in terms of hospital admissions compared to control group 1 and 2) is determined. This in turn is contrasted with the costs of the intervention. Finally, the net costs of the intervention (intervention costs minus costs of avoided hospital admissions) are compared with the intervention effects in terms of quality of life of the patients and reduction of the caregivers’ burden. To show the inference for the individual cost-effectiveness relations, a 95% confidence interval is calculated using Fieller’s Theorem (REF [74]).

In addition, the precise costs are evaluated for the subgroup for which individual data of the participating health insurance companies is available.

#### Qualitative evaluation

The interviews conducted in the process evaluation will be recorded and transcribed with digital audio recorders. The evaluation of the qualitative data is based on the qualitative content analysis procedure according to Mayring [75].

## Discussion

Due to their complex medical and psychosocial needs, PwD and their caregivers in particular require patient-centered, multiprofessional and networked care. To achieve needs-based medical and psychosocial care for PwD and their caregivers, a transformation of GP-based primary care is urgently needed. This transformation should provide timely dementia diagnoses, structured assessment of the actual medical and psychosocial needs of PwD and caregivers, stress-preventive support, and avoidance of obstructive polypharmacy. Another important aspect is the implementation of outpatient crisis interventions to avoid unnecessary hospital admissions.

The objective of the present study is to describe the evaluation of the complex GP-based DemStepCare intervention that aims to meet the transformation targets mentioned above to improve primary dementia care. The key feature of DemStepCare is the needs-based treatment of PwD, which is ensured by case management. Communication between the various parties involved is facilitated by an electronic case record. DemStepCare is accompanied by a formative process evaluation and a summative evaluation.

### Limitations

The progress of dementia will vary greatly among the participants. Therefore, some patients may not be able to complete the questionnaires, which might lead to a selective loss of data. Although in many cases we will have access to patient data from caregivers, it may be that they are not able to give an evaluation without bias. Past research has found differences between the evaluation of the quality of life of PwD made by PwD themselves and by caregivers, with caregivers usually evaluating the patients’ quality of life more negatively [76–78]. Therefore, proxy assessments in this area cannot be seen as a substitute for self-assessments and must be interpreted with caution.

Even though GPs are a central part of DemStepCare, only a small percentage of GPs are able to be personally interviewed. Of course, all GPs are covered by the panel survey. Nevertheless, it would be interesting to interview this target group individually to obtain information that is more specific.

Other weaknesses concern the recruitment of and by GPs: As GPs participate in the program voluntarily, all participating GPs are likely to be particularly interested in dementia. So there is a certain selection bias, leaving it unclear how the program works for less committed GPs. Since PwD in control group 1 benefit only marginally from DemStepCare, GPs in control group 1 may be less motivated to include patients than GPs in the intervention group. This could lead to different numbers of patients being recruited per group.

### Strengths

One of the strengths of the study is the multimodal approach that involves various actors in DemStepCare. Qualitative and quantitative data will be collected, the latter supplemented by routine data from the participating health insurance companies. By adopting a multimodal approach, we follow the recommendations for the evaluation of complex interventions [79].

Another methodological strength is the cluster randomized design and the use of two control groups. In control group 1 we are able to exploit all the possibilities of data collection that we also use in the intervention group. Control group 2, on the other hand, provides us with data that reflects current usual dementia care in Germany.

In terms of content, the importance of the caregivers is particularly noteworthy. While two of our three primary endpoints are patient-related, the third relates to the caregivers’ experienced burdens. In addition, other secondary endpoints relate to the caregivers. Family caregivers are key players in primary dementia care [4]. Their health is associated with the stability of the care situation [44]. In DemStepCare, this central aspect is taken into account and considered in the evaluation.

In summary, the DemStepCare intervention brings together promising aspects which are potentially useful in the primary care of PwD and their caregivers. A comprehensive process and outcome evaluation will help to assess the effectiveness of the intervention.

## Data Availability

The study data will not be publicly available as it is potentially identifying/confidential patient data.

## Abbreviations

GP: General practitioner
PwD: People with dementia
